# The anticancer activity of bovine lactoferrin is reduced by deglycosylation and it follows a different pathway in cervix and colon cancer cells

**DOI:** 10.1002/fsn3.4020

**Published:** 2024-03-07

**Authors:** Diana A. Ramírez‐Sánchez, Adrián Canizalez‐Román, Nidia León‐Sicairos, Gaspar Pérez Martínez

**Affiliations:** ^1^ Programa Regional de Noroeste para el Doctorado en Biotecnología Universidad Autónoma de Sinaloa Facultad de Ciencias Químico Biológicas Culiacan Mexico; ^2^ Unidad de Investigación, Facultad de Medicina Universidad Autónoma de Sinaloa Culiacan Mexico; ^3^ Servicios de Salud de Sinaloa Hospital de la Mujer Culiacan Mexico; ^4^ Servicios de Salud de Sinaloa, Departamento de Investigación del Hospital Pediátrico de Sinaloa Culiacan Mexico; ^5^ Consejo Superior de Investigaciones Cientificas Instituto de Agroquímica y Tecnología de Alimentos Paterna Spain

**Keywords:** Akt signal transduction, bovine lactoferrin, cancer cells inhibition, cell surface adhesion, glycosylation, internalization

## Abstract

Bovine lactoferrin (bLF) is a glycosylated protein with purported beneficial properties. The aim of this work was to determine the role of bLF glycosylation in the adhesion, internalization, and growth inhibition of cancer cells. The viability of cervix (HeLa) and colon (Caco‐2) cancer cells (MTT assay and epifluorescence microscopy) was inhibited by bLF, while deglycosylated bLF (bLFdeg) had no effect. Adhesion to cell surfaces was quantified by immunofluorescence assay and showed that bLF was able to bind more efficiently to both cell lines than bLFdeg. Microscopic observations indicated that bLF glycosylation favored bLF binding to epithelial cells and that it was endocytosed through caveolin‐1‐mediated internalization. In addition, the mechanism of action of bLF on cancer cell proliferation was investigated by determining the amount of phosphorylated intermediates of signaling pathways such as epidermal growth factor receptor (EGFR), extracellular signal‐regulated kinase (ERK) and protein kinase B (known as Akt). Chemoluminescence immunoassay of phosphorylated intermediates showed that bLF inhibited Akt phosphorylation, consistent with its growth inhibiting activity. This assay also indicated that the bLF receptor/signaling pathways may be different in the two cell lines, Caco‐2 and HeLa. This work confirmed the effect of glycosylated bLF in inhibiting cancer cell growth and that glycosylation is required for optimal surface adhesion, internalization, and inhibition of the ERK/Akt pathway of cell proliferation through glycosylated cell surface receptors.

## Graphical abstract

The native glycosylation state of bovine lactoferrin (bLF) is required for the inhibition of growth of cervix (HeLa) and colon (Caco‐2) cancer cells by blocking Akt phosphorylation. The response of Caco‐2 and HeLa cells to bLF involves different signal transduction intermediates possibly following different receptor/signaling pathways. bLF is endocytosed by caveolin‐1‐mediated internalization and glycan residues are essential for the interaction with both cell lines.

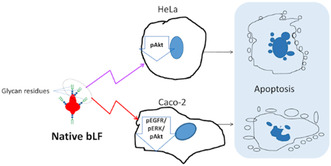

## 1 INTRODUCTION

Lactoferrin (LF) is an iron‐binding glycoprotein primarily found in milk and other body secretions, which has been consolidated as a remarkably beneficial protein for its antimicrobial (Acosta‐Smith et al., 2017), antiviral (Hu et al., 2021), antifungal (Manzoni et al., 2012), anti‐inflammatory (Goulding et al., 2021), and immune stimulatory function (Siqueiros‐Cendón et al., 2014). Bovine lactoferrin (bLF) (80 kDa) has in vitro and in vivo anticancer activity against several types of cancer (Chea et al., 2018; Gibbons et al., 2015; Haney et al., 2012; Li et al., 2017; Luzi et al., 2017; Rocha et al., 2022; Sun et al., 2012; Wolf et al., 2003; Zhang et al., 2015), and it induced a reduction in colorectal cancer polyps, improvement in patient quality of life, and reduction in toxic side effects of chemotherapy drugs (Kozu et al., 2009; Moastafa et al., 2014). This anticancer activity of bLF has been attributed to inhibition of cell viability by disruption of the cell membrane, induction of cell cycle arrest, apoptosis, or iron deprivation (Fujita et al., 2004; Gibbons et al., 2015; Li et al., 2011; Luzi et al., 2017; Ramírez‐Sánchez et al., 2021). Activation of EGFR is a prerequisite in several oncogenic processes, leading to the activation of cell proliferation signaling pathways such as the ERK/MAPK and AKT‐PI3K‐mTOR pathways (Edick et al., 2007; Wee & Wang, 2017). Testing the phosphorylation of intermediates in these pathways will provide a solid proof of concept supporting the activation or inhibition of cancer cell growth.

Like ovine and caprine LF, bLF has five N‐glycosylated asparagine residues at Asn233, Asn281, Asn368, Asn476, and Asn545, whereas human LF has only three N‐glycosylation sites, at Asn138, Asn479, and Asn624 (Karav et al., 2017). Research into the biochemical and structural role of bLF glycosylation has shown that glycosylation protects against proteolysis (O'Riordan et al., 2014; van Berkel et al., 1995) and deglycosylation reduces the iron binding capacity of hLF and bLF (O'Riordan et al., 2014). Deglycosylation of bLF can occur upon exposure to enzymes and microorganisms in the gastrointestinal environment, which are known to alter the biological activities of bLF; therefore, it would be most relevant to determine how deglycosylation might affect the activity of bLF (Nakamura‐Bencomo & Gutierrez, 2021). In fact, glycans are also involved in signaling, cellular recognition, and binding processes (Karav et al., 2017), as the glycosylation state of bLF has a differential effect on the stimulation of pattern recognition receptors (Figueroa‐Lozano et al., 2018), but its association with bLF anticancer mechanisms has not been explored. This study will show the relevance of bLF glycosylation on the inhibition of viability, surface binding, internalization, and modulation of signaling pathways in cervix and colon cancer cell lines, hence providing new insights into the mechanism of action of bLF anticancer activity.

## 2 MATERIALS AND METHODS

### 2.1 Reagents and cell cultures

Bovine lactoferrin (bLF) produced by Morinaga Milk Industry was donated by Milei GmbH (Germany), resuspended in sterilized H_2_O, incubated overnight at 4°C and harvested at 2,500 rpm for 10 min. The supernatant was filtrated and bLF was quantified according to the instructions of the Bicinchoninic Acid Protein Assay Kit (Sigma‐Aldrich). The purity of 98% bLF reported by the manufacturer was confirmed by SDS‐PAGE 7%. BLF from Sigma‐Aldrich (>85%) was also used in some experiments. Peptide N‐glycosidase F from *Flavobacterium meningosepticum* (PNGase F) (Sigma‐Aldrich) was used for enzymatic deglycosylation.

HeLa cells and Caco2 cells respectively derived from cervix and colon cancer were used. Both cell lines were maintained in DMEM, 10% SFB, and 1% penicillin/streptomycin (Gibco™). Caco2 medium was supplemented with 1% non‐essential amino acids (Gibco™), 1% sodium pyruvate (Gibco™), 1% HEPES (Gibco™), and 1% L‐glutamine 2 mM (Gibco™).

### 2.2 Protein deglycosylation

bLF was incubated with PNGase F for 72 h at 37°C to maximize deglycosylation. For each 100 μg of LF, 1 μL of PNGase F was used in 50 mM sodium phosphate buffer. Samples were diluted 1:10 in loading buffer, denatured at 95°C for 10 min, and visualized by 7% SDS‐PAGE with Coomassie Blue after staining. Images were captured on a photodocumenter (ProXima 16 Phi+, Isogen, Life Science).

### 2.3 Cell viability assay

To evaluate the inhibition of cell viability by native and deglycosylated bLF, the 3‐(4,5‐dimethylthiazol‐2‐yl)‐2,5‐diphenyltetrazolium bromide (MTT) assay was performed as described before (Ramírez‐Sánchez et al., 2021). Briefly, HeLa and Caco2 cells were inoculated at a density of 2 × 10^4^ cells per well in a 96‐well plate and incubated for 24 h at 37°C and 5% CO_2_. Cells were treated with bLF (native or deglycosylated) at concentrations of 1, 10, and 20 μM for 24 h, with a control of untreated cells. After treatment, the cells were washed with PBS and incubated with 100 μL MTT (5 μg/mL final concentration; Sigma‐Aldrich) for 4 h at 37°C in the dark. The same volume of solvent (1N HCl isopropanol) was then added and incubated for 30 min or 1 h until the formazan crystals were dissolved and cell viability was measured at 570 nm in a POLARstar plate reader (POLARstar Omega, BMG Labtech). The percentage of cell viability was calculated by dividing the optical density (OD) of each treatment by the OD of the untreated control cells and multiplying by 100. Experiments were performed in triplicate with three independent replicates in each assay.

### 2.4 Quantification of adhesion to cell culture surface

Adhesion of native (bLF) and deglycosylated bLF (bLFdeg) to the cell surface was quantified by an adaptation of the fluorescence activation cell sorting (FACS) method (Greenfield, 2022). HeLa and Caco2 cells were cultured in black 96‐well plates at a density of 3.5 × 10^4^ and 5 × 10^4^ cells per well, respectively, and were incubated at 37°C in 5% CO_2_, HeLa was incubated for 24 h and Caco2 for 48 h. Then, cell layers were washed with PBS and they were incubated with 10 μM bLF and bLFdeg in DMEM, without antibiotics or supplements, for 10 min and 1 h at 37°C. Control wells without bLF or bLFdeg were also included. Cells were washed three times with PBS, fixed with 100 μL of 4% PFA for 10 min at room temperature in the dark, washed three times, and incubated with 50 μL of blocking solution (4% BSA in PBS) for 1 h at room temperature. After that, cells were treated with 50 μL of rabbit anti‐LF primary antibody (Sigma Aldrich) at 1:400 in 1% BSA in PBS. Then samples were washed three times with PBS and after this, they were incubated with 50 μL of Alexa Fluor 594‐conjugated anti‐rabbit secondary antibody (Thermofisher Scientific) at 1:500 in 1% BSA in PBS for 1 h at room temperature. After that, they were washed three times with PBS and 20 μL of PBS was added for the measurement of fluorescence at 540/610 nm using a POLARstar Omega microplate reader (BMG LABTECH).

### 2.5 Determination of phosphorylated intermediates in signaling pathways

The levels of phosphorylated intermediates of different growth/proliferation signaling pathways were quantified by antibody capture on permeabilized cells—intracellular binding and chemoluminescence method described by others (Greenfield, 2022). HeLa and Caco2 cells were seeded in 96‐well white plates for chemoluminescence at a density of 3.5 × 10^4^ and 5 × 10^4^ cells per well, respectively, in DMEM medium supplemented as required for each cell line. HeLa cells were incubated for 24 h and Caco2 for 48 h, both at 37°C, 5% CO_2_. The cells were then subjected to a period of cell starvation in DMEM medium without FBS or antibiotics for 24 h before stimulation. The cells were washed twice with PBS and incubated with 10 μM bLF and bLFdeg for 10 min and 1 h, respectively. As a positive control or reference, 100 ng/mL EGF was used and untreated cells as a negative control. Cells were washed and fixed with 50 μL of 4% PFA for 10 min at room temperature in the dark, and they were subsequently permeabilized with 50 μL of 0.2% Triton X‐100 for 10 min and then blocked with 50 μL of blocking solution (4% BSA in PBS) for 1 h at room temperature. Proteins detected with specific antibodies were: phosphorylated Akt (pAKT), phosphorylated MAP kinases ERK1 and ERK2 (pERK 1/2), phosphorylated NF‐κB p65, and phosphorylated EGF receptor (pEGFR). The blocking solution was then removed and 40 μL of primary anti‐pEGFR, anti‐pAKT, anti‐NF‐κB p65, or anti‐pERK1/2 antibody (Table 1) was added at a 1:400 ratio in 1% BSA in PBS, and the plates incubated overnight at 4°C. After that, the cells were washed and incubated with 50 μL of horseradish peroxidase‐conjugated anti‐rabbit secondary antibody for 1 h. The reaction was detected using 20 μL per well of the detection reagent mixture (solution A and B 1:1) from the ECL Prime Western Blotting Detection Reagents Kit (GE Healthcare, Cytiva). The chemoluminescence reaction was quantified using the POLARstar Omega plate reader (BMG LABTECH), detecting emission at 460 nm. The following table details the different antibodies used (Table 1).

**TABLE 1:** Antibodies used for the detection of phosphorylated proteins related to cell growth/proliferation or apoptosis.

Antibody	Type	Molecular weight (kDa)	Manufacturer
*Phospho‐Akt (Ser473)*	Primary Policlonal from rabbit	60	Cat. 9271S, Cell Signaling Technology
*P p44/p42 MAPK (ERK 1/2) (*Thr202/Tyr204)	Primary Policlonal from rabbit	42–44	Cat. 9101S, Cell Signaling Technology
*p‐EGF Receptor* (Tyr1068) *(Y1068)*	Primary Policlonal from rabbit	175	Cat. 2234S, Cell Signaling Technology
*Phospho‐NF‐ҡB p65 (Ser536)*	Primary Policlonal from rabbit	65	Cat. 3031S, Cell Signaling Technology
*Anti‐rabbit IgG horseradish peroxidase conjugate*	Secondary	–	GE Healthcare

### 2.6 Morphological changes and adhesion visualization of native and deglycosylated bLF

HeLa and Caco2 cells were seeded on sterile glass coverslips in 24‐well plates at a density of 2 × 10^5^ and 3 × 10^5^, respectively, and incubated for 24 h for HeLa and 48 h for Caco2 to >80% confluence. Cells were washed with PBS and incubated with 10 μM bLF or bLFdeg in DMEM medium without FBS and antibiotics for 24 h at 37°C and 5% CO_2_. Control wells without bLF or bLFdeg were also included. The cells were washed, fixed with 200 μL of 4% paraformaldehyde for 10 min at room temperature in the dark, and they were stained with phalloidin‐TRITC (Sigma Aldrich) 1:250 in PBS for 20 min and with DAPI (Sigma Aldrich) 1:200 in PBS for 10 min. The coverslips were mounted on slides with 7 μL of ProLong Gold (Thermo Fisher Scientific) anti‐discolor mounting solution. A Nikon Eclipse E90i fluorescence microscope (Nikon Corporation, Japan) was used for visualization. Images were captured and processed with the Nis Elements BR 3.2 program (Nikon Corporation, Japan) using epifluorescence filters: for blue, DAPI 340‐380/435‐475 and; for red, G‐2a 510‐560/565‐ 615, excitation and emission, respectively.

For visualization of bLF adhesion to cells after washing with PBS, cells were incubated with bLF and bLFdeg medium without SFB and antibiotics for 10 min at 37°C and 5% CO_2_, and then cells were fixed with 4% paraformaldehyde as before. To detect bLF, the primary antibody rabbit anti‐bLF (Sigma‐Aldrich) was used at a ratio of 1:400 in 1% BSA, incubated for 1 h. The secondary antibody anti‐rabbit Alexa Fluor 488 (Invitrogen) was used at a ratio of 1:500 in 1% BSA. Cells were re‐fixed and stained with WGA‐Alexa Fluor 594 1:400 (ThermoFisher Scientific) for 15 min. Cell nuclei were stained with DAPI (Sigma‐Aldrich) 1:200 for 10 min in the dark. Samples were mounted on slides and visualized as described. Images were acquired and processed using Nis Elements BR 3.2 software (Nikon Corporation, Japan) and DAPI 340‐380/435‐475 (blue), FITC 465‐495/515‐555 (green), and G‐2a 510‐560/565‐615 (red) filters.

### 2.7 Internalization and colocalization with clathrin and caveolin

As described before, HeLa and Caco2 cells were seeded on sterile glass coverslips and allowed to reach >80% confluence. Most of the staining and microscopic procedures were carried out as before adapted to the purpose of this work (Ramírez‐Sánchez et al., 2021). Cells were treated with 10 μM bLF and bLFdeg in DMEM medium without FBS and antibiotics for 10 min and 1 h at 37°C and 5% CO_2_, and coverslips without bLF and bLFdeg were also included as control. The cells were fixed with 200 μL of 4% PFA for 10 min at room temperature in the dark and permeabilized with 200 μL of 0.2% Triton for 10 min. Then cells were blocked with 4% BSA blocking solution (Sigma Aldrich) in PBS for 1 h, they were incubated with rabbit anti‐bLF primary antibody (Sigma Aldrich) at 1:400 in 1% BSA in PBS for 1 h, and with goat anti‐rabbit secondary antibody conjugated to Alexa Fluor 594 (Cat. A‐11012, Thermofisher Scientific) at a 1:500 ratio in 1% BSA in PBS for 1 h. Again, the cells were blocked and incubated with primary mouse monoclonal anti‐clathrin and anti‐caveolin 1 antibodies (ThermoFisher Scientific) at 1:400 in 1% BSA in PBS for 1 h. Then, the cells were incubated with goat anti‐mouse secondary antibody (Sigma Aldrich) for 1 h. Finally, the cells were fixed with 200 μL of 4% paraformaldehyde for 10 min and stained with DAPI (1 mg/mL) at 1:200 for 10 min. The coverslips were mounted on slides with 7 μL of antifade solution ProLong Gold (ThermoFisher Scientific). The Nikon Eclipse E90i fluorescence microscope (Nikon Corporation, Japan) was used for visualization. Images were acquired and processed using the Nis Elements BR 3.2 program (Nikon Corporation, Japan) with the following filters: for blue, DAPI 340‐380/435‐475; for green, FITC 465‐495/515‐555; for red, G‐2a 510‐560/565‐615, excitation and emission, respectively.

### 2.8 Statistical methods

Experiments were performed three times with four replicates for each condition and data were analyzed as previously (Ramírez‐Sánchez et al., 2021) with IBM SPSS Statistics version 23 and GraphPad Prism7. Analysis of variance was performed using a mixed analysis of variance with two fixed factors (cell line, bacteria) and two random factors (day and well). The Bonferroni or Tamhane multiple comparison test was used to compare treatments, with a *p* ≤ .05 considered significant (Ramírez‐Sánchez et al., 2021).

## 3 RESULTS

### 3.1 Effect of bLF deglycosylation on the inhibition of cervix and colon cancer cells

**FIGURE 1:**
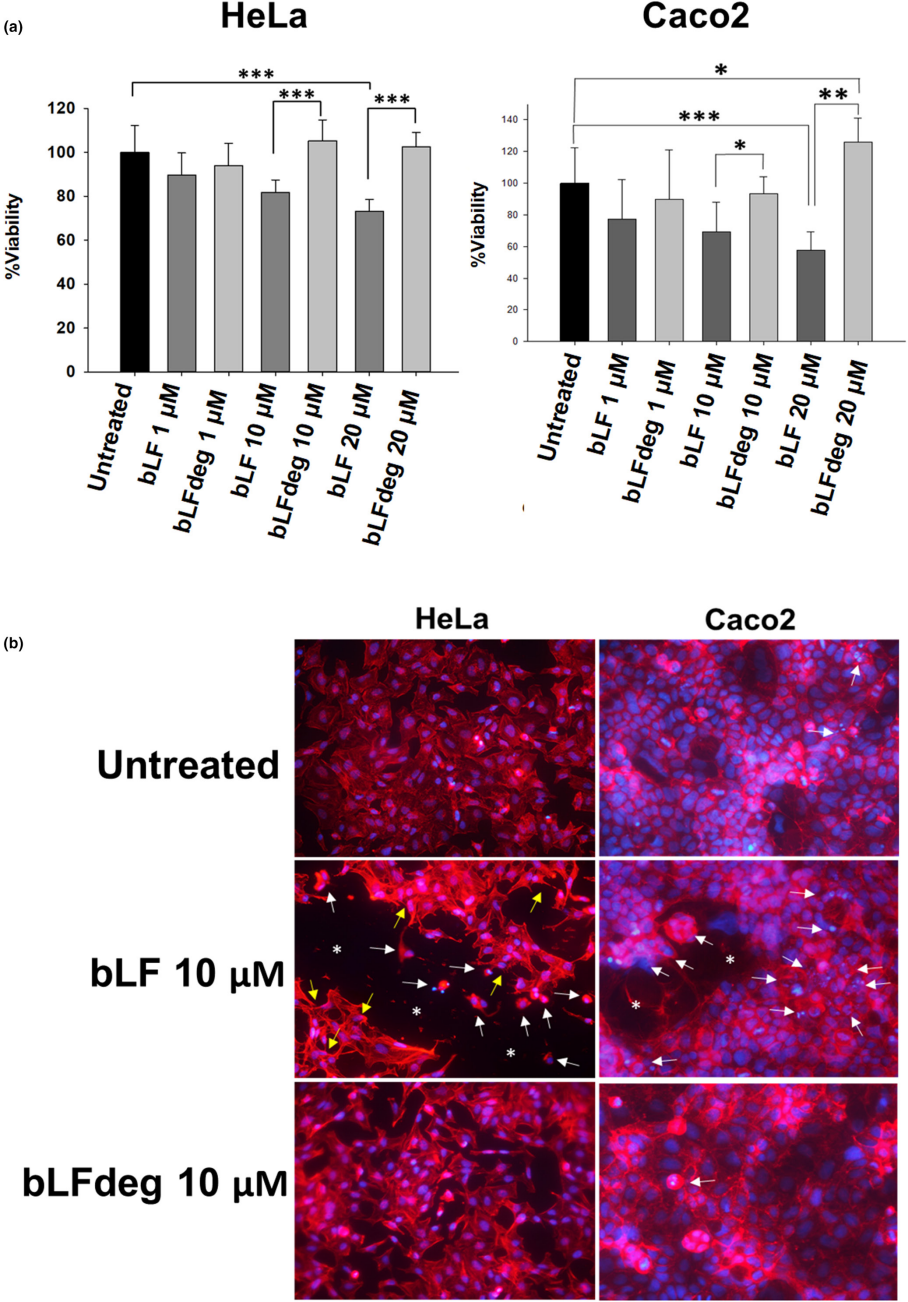
Effect of bLF deglycosylation on viability and morphology of HeLa and Caco2 cell lines. (a) Cell viability of HeLa and Caco2 cells determined by MTT assays after their treatment with bLF or bLFdeg (1, 10, and 20 μM) for 24 h on. Untreated cells were used as control. Data were obtained after five replicates in three independent experiments. The graphs show the means and vertical bars of the standard deviation (SD). Data significance was determined by student‐t. *p ≤ .05, **p ≤ .01, ***p ≤ .001. (b) Morphological changes observed in HeLa and Caco2 cells after the treatment with 10 μM bLF and 10 μM bLFdeg for 24 h, using as control untreated cells. Preparations of cell monolayers were visualized by fluorescence microscopy. Actin cytoskeleton was stained with phalloidin‐TRITC (red) and nuclei with DAPI (blue). White arrows indicate cell shrinkage and rounding, nuclear compaction or condensation, and yellow arrows show alterations in the cytoskeleton organization and normal cell shape, all of which are morphological changes indicative of typical apoptotic processes.

The inhibitory activity of bLF and bLFdeg on cervix (HeLa) and colon (Caco2) cells was determined using MTT assays. Native bLF reduced the viability of HeLa and Caco2 in a concentration‐dependent manner (Figure 1a), and significant inhibition was reached at 20 μM, in both Hela (73% viability) and Caco2 (57% viability) compared to the control. No detectable effect was observed with bLFdeg (>90% viability at all concentrations tested). Fluorescence microscopy revealed morphological changes occurring in HeLa and Caco2 cells. The images showed that bLF induced morphological changes similar to apoptosis in both cell lines (HeLa and Caco2), such as cell shrinkage, actin cytoskeleton disruption, rounding, and/or nuclear condensation, leading to significant cell depletion, while no differences were observed between untreated and bLFdeg treated cells (Figure 1b).

### 3.2 Effect of deglycosylation on cell surface adhesion

**FIGURE 2:**
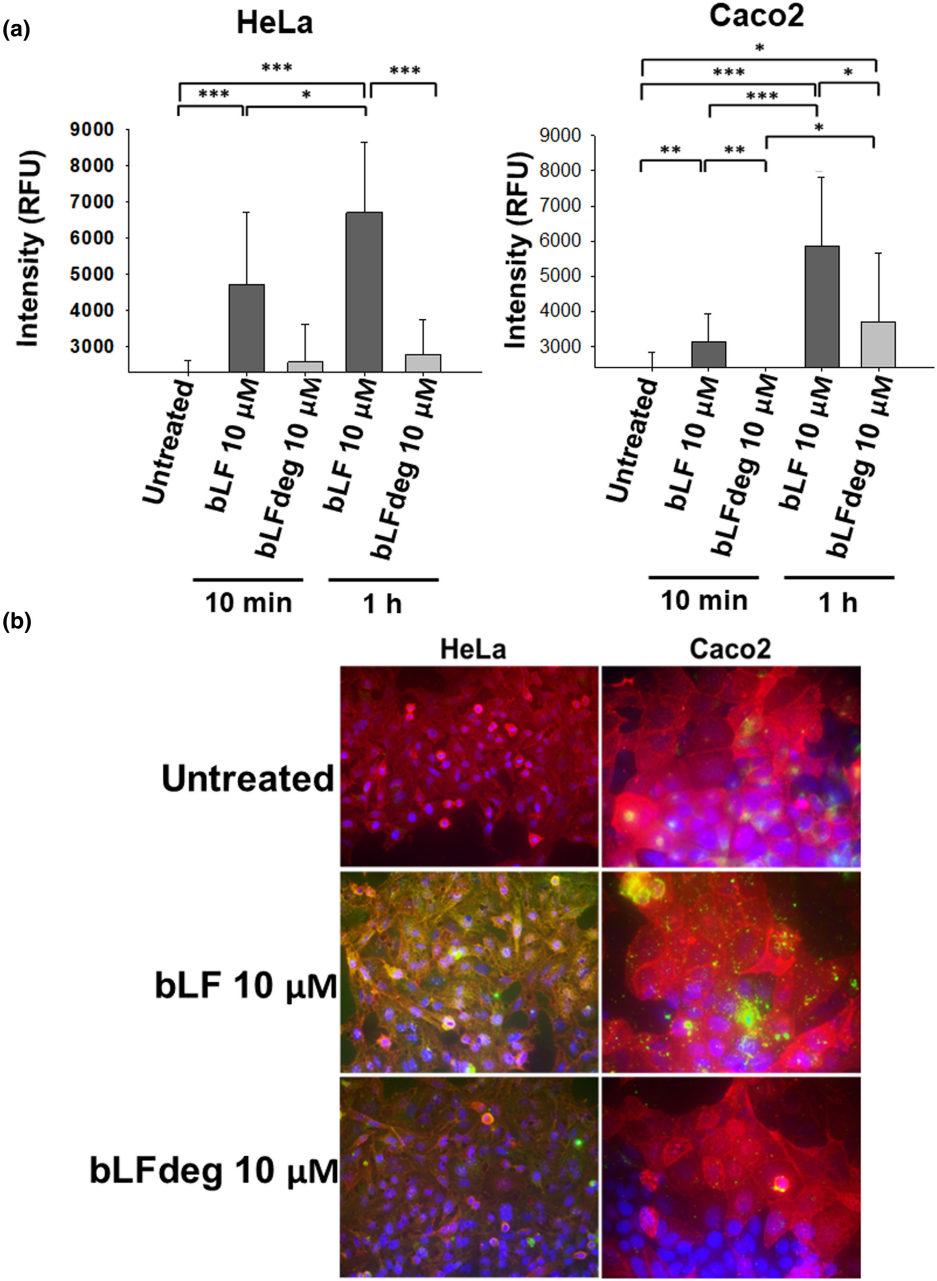
Deglycosylation of bLF reduces its binding capacity to HeLa and Caco2 cells. (a) HeLa and Caco2 cells were treated with bLF and bLFdeg during 10 min and 1 h, and untreated cells were used as control. Assays were performed with three replicates and in three independent experiments. The average and standard deviation (SD) are shown. Significance was tested by t‐student and significance levels are indicated: *p ≤ .05, **p ≤ .01, ***p ≤ .001. (b) bLF and bLFdeg binding to HeLa and Caco2 were visualized by immunofluorescence (green). Cells were stained with WGA 564 (cell membranes in red) and DAPI (nuclei in blue).

A reliable quantification of bLF and bLFdeg adhesion to the cell surface was developed as a first step to assess the interaction of bLF with cultured cells. For this purpose, a fluorescence spectrophotometric method was implemented in which bLF attached to the cell surface of epithelial cells was detected with a rabbit anti‐bLF antibody, which could be quantified with a fluorescent secondary antibody (anti‐rabbit labeled with Alexa Fluor 594). High fluorescence of the cell cultures indicated a high level of adhesion of bLF to both HeLa and Caco2 cells (Figure 2a), with a significant increase in adhesion from 10 min to 1 h. Low fluorescence signal of bLFdeg indicated low adhesion, similar to the control of untreated cells in both cell lines. These results strongly suggest that bLF glycans are essential for binding to cultured cell surfaces. The binding of bLF and bLFdeg was also visualized by immunofluorescence microscopy, where some differences between bLF and bLFdeg binding to HeLa and Caco2 cells could be observed (Figure 2b). In HeLa cells, bLF labeled the cell surface uniformly, in a pattern similar to WGA594, a wheat germ agglutinin (WGA)‐coupled fluorochrome with affinity for specific surface glycans. In contrast, the binding pattern of bLF to Caco2 cells was concentrated in intense spots or small areas on the cell surface, suggesting differences in the topology of the binding sites/receptors between the two cell lines. The importance of glycosylation of putative receptors on the cell surface was also investigated in both cell lines by treating them with peptide‐N‐glycosidase F (PNGase F) using the fluorescence spectrophotometric method. The results showed that surface deglycosylation had a greater effect on the adhesion of bLF to HeLa cells (Supporting information Figure S1).

### 3.3 Internalization of bLF in HeLa and Caco2 cells

**FIGURE 3:**
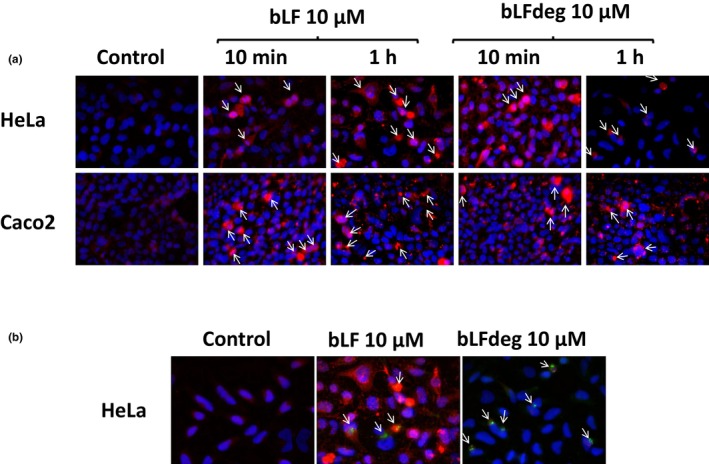
Internalization of bLF and bLFdeg in HeLa and Caco2. (a) HeLa and Caco2 cells were treated with bLF and bLFdeg during 10 min and 1 h. Untreated cells were used as control. Preparations show nuclei stained in blue (DAPI) and bLF (native and deglycosylated) in red with a secondary antibody coupled to AlexaFluor 594. (b) Colocalization of bLF with caveolin‐1 in HeLa cells. HeLa cells were treated with native and deglycosylated bLF during 1 h. Untreated cells were used as control. Association of bLF (native and/or deglycosylated) was visualized by staining bLF (native and deglycosylated) in red with a secondary antibody AlexaFluor 594 and caveolin‐1 in green with a secondary antibody AlexaFluor 488. As above, cell nuclei were stained by blue (DAPI).

After treatment of HeLa and Caco2 cells with bLF or bLFdeg for 10 min and 1 h, bLF was visualized by epifluorescence microscopy using labeled antibodies (Figure 3a). Higher fluorescence intensity indicated that bLF adhered and was internalized to a greater extent than bLFdeg (white arrows) in both cell lines. Differences in binding between bLF and bLFdeg can be observed after 10 min and 1 h of incubation. Apparent internalization was interpreted by the presence of bLF‐containing vesicles. Internalized bLF and bLFdeg were located in the nuclear periphery, also in mitotic cells. Deglycosylation of bLF significantly reduced its adherence and thus its internalization potential. After internalization, bLF and bLFdeg were located at the nuclear periphery. To confirm that bLF was internalized into cell vesicles via clathrin‐ or caveolin‐dependent endocytosis, colocalization with clathrin and caveolin was examined by immunohistochemistry after 10 min or 1 h in HeLa cells. No apparent co‐staining of bLF and clathrin was observed under our experimental conditions (images not shown), but positive colocalization of bLF or bLFdeg with caveolin‐1 was observed, suggesting caveolin‐dependent endocytosis (Figure 3b, white arrows).

### 3.4 bLF inhibits signaling pathways involved in cell proliferation

**FIGURE 4:**
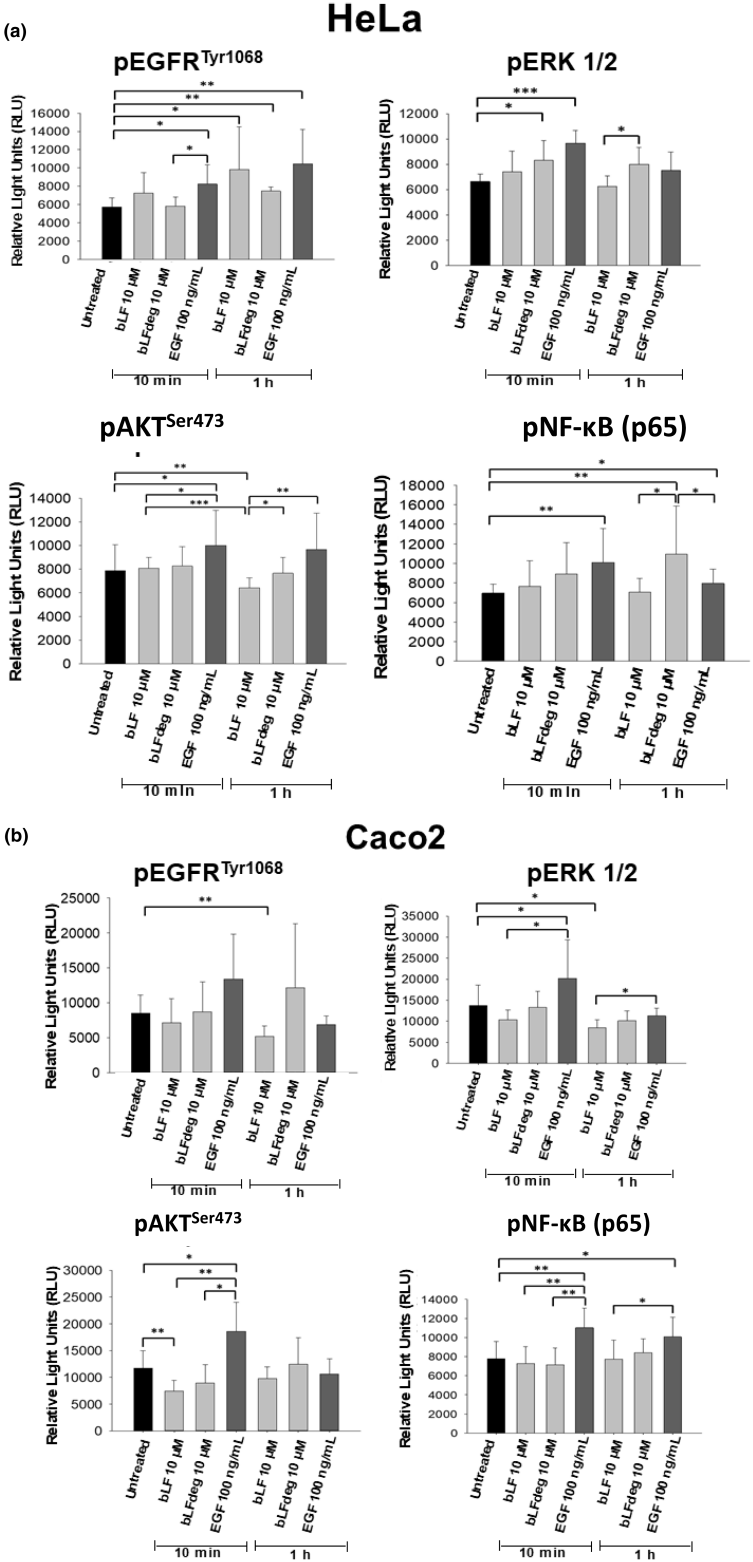
Effect of bLF and bLFdeg on phosphorylation of intermediates in signaling pathways. Levels of phosphorylated proteins: EGFR, ERK 1/2, AKT, and p65 (NF‐ĸβ) determined by chemoluminescence immunoassay (see text). Controls of untreated cells or stimulated with EGF 100 ng/mL were included. (a) Cervix HeLa cells treated with bLF and bLFdeg 10 μM for 10 min and 1 h. (b) Colon adenocarcinoma Caco2 cells were treated with bLF and bLFdeg 10 μM for 10 min and 1 h. Assays were carried out with four replicates and in three independent experiments. The average and standard deviation (SD) are shown in the graph. Student *t*‐test was used to determine significance between treatments (*p ≤ .05, **p ≤ .01, ***p ≤ .001).

The participation of bLF, and the effect of bLF deglycosylation, in activation or suppression of signaling pathways of cell growth and proliferation mediated by phospho‐EGFR (pEGFR), phospho‐Akt (pAKT), phospho ERK1/2 (pERK), and p65 (pNF‐ĸB) in HeLa and Caco2 were examined by the quantification of phosphorylated intermediates by chemiluminescence immunoassay. In HeLa cells, EGFR phosphorylation (pEGFR^Tyr1068^) was stimulated by bLF and bLFdeg treatment (Figure 4a). bLFdeg stimulated pERK1/2 after a short time of incubation (10 min) and also increased p65 (pNF‐ĸβ). A reduction of pAKT^Ser473^ was observed after 1 h by bLF treatment. These data suggest that PI3K‐AKT, MAPK/ERK, and NF‐ĸB pathways are probably inhibited or stimulated, respectively, by bLF and bLFdeg in HeLa cells, as will be discussed below. In contrast, in Caco2 (Figure 4b), bLF decreased pEGFR, pERK1/2, and pAKT (after 10 min), the key proteins in PI3K/AKT and MAPK/ERK pathways. This indicated that bLF clearly inhibited these signaling pathways, which could lead to cell growth and proliferation, while bLFdeg had no significant effect in Caco2. The pNF‐ĸB (p65) was unaltered by bLF. For a quick overview, a table is also attached showing the significant effects of bLF and bLFdeg treatments with respect to controls (Table 2).

**TABLE 2:** Table summarizing results from the assay of signaling pathways intermediate phosphorylation.

Phosphorylated marker	Effect on phosphorylation (vs. control)	
	HeLa	Caco2
pEGFR^Tyr1068^	bLF ↑ bLFdeg ↑	bLF ↓ bLFdeg‐
pERK1/2	bLF‐ bLFdeg ↑	bLF ↓ bLFdeg‐
pAKT^Ser473^	bLF ↓ bLFdeg‐	bLF ↓ bLFdeg‐
pNF‐κB(p65)	bLF‐ bLFdeg ↑	bLF‐ bLFdeg‐

*Note*: The table indicates significant differences of samples treated with bLF and bLFdeg with respect to controls. Arrows indicate a significant effect after 10 min incubation.

## 4 DISCUSSION

Bovine lactoferrin (bLF) is a very abundant protein in cow's milk with claimed health effects (Artym et al., 2021; Hassoun & Sivamani, 2017; Kaczyńska et al., 2023; Miyakawa et al., 2023). The potential anticancer effect of bLF was described some time ago, as oral administration of bLF specifically inhibited the development of colon, bladder, esophagus, and lung tumors in rats at the post‐initiation stage, but the molecular mechanisms are yet to be determined (Masuda et al., 2000; Tsuda et al., 2000; Ushida et al., 1999).

In glycosylated proteins, glycans protect against proteolytic degradation and play a fundamental role in various functions, such as immunogenicity, activation of signaling pathways, and cell adhesion (Karav et al., 2017). In the case of bLF, its deglycosylation affects some of its biological activities (van Berkel et al., 1995), but there are few studies that have investigated the role of structural glycans in the beneficial effects of bLF (Figueroa‐Lozano et al., 2020). This work aimed to study the impact of glycosylation in the anticancer activity of bLF by comparing its inhibitory activity with that of deglycosylated bLF (bLFdeg) against cervix (HeLa) and colon (Caco2). Indeed, bLF reduced the viability of HeLa and Caco2 cells in a concentration‐dependent manner and induced observable cell damage and morphological changes, hence confirming the anticancer activity of bLF shown in previous reports (Luzi et al., 2017; Ma et al., 2013; Ramírez‐Sánchez et al., 2021), and deglycosylation had a dramatic effect on the ability of bLF to adhere to the cell surface of both Hela and Caco‐2 cells.

Interestingly, binding of bLF was significantly reduced by surface deglycosylation of Hela cells, suggesting that binding of bLF to HeLa cells requires a greater interaction with surface glycoproteins than in Caco2, stressing the fact that receptors and subsequent induction of signaling may be different in the two cell lines. A number of proteins have been reported as LF receptors in different cell lines and tissues such as CD14, intelectin‐1 (omentin‐1), Toll‐like receptor 2 and 4 (TLR2, TLR4), and cytokine receptor 4 (CXCR4) (see for review (Kell et al., 2020)). LfR was identified as a specific receptor of human LF that associates with AP2, which mediates internalization via clathrin endocytosis to stimulate ERK signaling (Jiang et al., 2011). The low‐density lipoprotein receptor‐related protein (LRP‐1) is a major endocytic receptor known to bind more than 35 ligands with signaling properties (Etique et al., 2013), and different evidences suggest that LRP‐1 could be a likely bLF receptor in different models (Chea et al., 2023; Takayama et al., 2003). After binding to LRP‐1, bLF promoted the phosphorylation of Akt1 and extracellular signal‐regulated protein kinase (ERK1/2); an induction process that requires iron‐saturated bLF (holo‐LF) (Ryu et al., 2017). LF also binds to extracellular matrix and surface‐bound heparan sulfate proteoglycans (HSPGs) (van Berkel et al., 1997), which has been associated to numerous health effects, particularly to the anti‐viral activity of LF (Hu et al., 2021).

Internalization of bLF was mainly observed in the periphery of the nucleus of both cell lines, HeLa and Caco2, which is consistent with the reported location of hLF and bLF near the nucleus (Ashida et al., 2004; Jiang et al., 2011; Suzuki et al., 2008) where their modulation of gene expression (Cao et al., 2023). As mentioned above, human LF is internalized via clathrin‐dependent endocytosis, and also bLF in THP‐1 human macrophage‐like cells (Florian et al., 2012) and in Caco2 (Jiang et al., 2011). Here, caveolin‐dependent endocytosis of bLF and bLFdeg was observed, indicating that the mechanism of internalization is independent of the glycosylation state of bLF in HeLa cell cultures. Internalization of bLF by caveolin‐covered lipid rafts or vesicles has not been reported, its endocytosis would still require binding to LRP1 (Grey et al., 2004; Zhang et al., 2004). This represents some advance in the intake mechanism of bLF, but further studies are still required to fully define the complete mechanism of endocytosis of bLF in HeLa as well as in Caco2.

In HeLa bLF primarily induced apoptosis and autophagy‐related protein cells by upregulating p53 and cleaved‐caspase‐3, also reducing the expression of Bcl‐2, an antiapoptotic protein (Shu et al., 2023). Also in the intestinal cell line HT‐29, bLF induced apoptosis (caspase3/7 activity) and regulated gene expression through p53 signaling leading to cell death (Jiang & Lönnerdal, 2017). In endothelial cells, bLF inhibited proliferation, migration, and tube formation while increasing apoptosis by downregulation of VEGF‐A, VEGF receptor (VEGFR) and HIF‐1α via reducing p‐p65 binding to TRAF6 (Ayuningtyas et al., 2023), and this receptor was also inhibited by LF‐Se nanoparticles in MCF‐7, HepG‐2, and Caco‐2 cells (El‐Fakharany et al., 2023). Finally, in different metastatic cell lines, bLF downregulatd PI3K, and AKT or p‐AKT and inhibited glycolysis by interaction with V‐ATPase at the plasma membrane lipid rafts (Santos‐Pereira et al., 2022). Here, phosphorylation of intermediates such as EGFR (pEGFR), Akt (pAKT), ERK1/2 (pERK), and p65 (pNF‐ĸB) was determined in HeLa and Caco2 cells. They participate in key signaling pathways mainly related to cell survival and proliferation, but also to endocytosis, cytoskeletal rearrangement, and inflammation. In HeLa, bLF increased pEGFR and decreased pAKT, while bLFdeg also activated EGFR, ERK, and NF‐κB, suggesting induction of cell growth and a pro‐inflammatory state via ERK/MAPK activation (Jijon et al., 2012), possibly related to the lack of effect on the reduction in cell viability by bLFdeg. In contrast, Caco2 showed the canonical inactivation of EGFR, ERK1/2 and Akt, as would be expected from stimuli that inhibit growth (Huang et al., 2019) and/or induce apoptosis (Ivanov & Hei, 2005; Sarkar et al., 2010). Independently of which would be the receptor of bLF, here, bLF repressed EGFR and the ERK/Akt pathway in Caco2. If LRP‐1 is the bLF receptor, its stimulation by bLF would lead to the activation of Scr kinases activating tyrosine kinase (Trk) receptors, such as EGFR, that in turn would increase pERK and pAKT (Mao et al., 2017; Shi et al., 2009). In HeLa, response to bLF was complex where the increase of pEGFR and reduction of pAKT was probably connected to the observed growth inhibition of HeLa by bLF. In this case, the increase of pEGFR could be due to stimulation through LRP‐1, and bLFdeg increased ERK1/2 which could be consequence of activation of MAPK/ERK pathway for growth and differentiation. But the opposite effect was observed in Caco2, where bLF repressed EGFR and the ERK/Akt pathway in Caco2. The EGFR signaling pathway involves a complex map of interactions with numerous signaling pathways (Oda et al., 2005). EGFR is linked to the ERK‐MAPK pathway through two adaptor proteins, GRB2 and SHC (Shaul & Seger, 2007), and other kinase cascades leading to MEK and the terminal MAPK, ERK, through RAF proteins (Katz et al., 2007). Due to the great relevance of the EGFR signaling pathway future studies should consider the involvement of these kinases and other proteins mentioned in the signals induced by bLF in different cell lines.

As mentioned above, cell surface (N‐)deglycosylation inactivated bLF induction, especially in HeLa cells. Like other surface proteins and receptors, LRP‐1 has a large number of glycosylation sites (Pedersen et al., 2014), also N‐glycosylation of the EGFR extracellular domain is essential for its binding and activation by growth factors (Azimzadeh Irani et al., 2017). In Caco2, (N‐)deglycosylation of the cell surface had little effect (not significant) on bLF adhesion, but in HeLa, differences in bLF adhesion after (N‐)deglycosylation were remarkable, supporting the interaction of bLF with a (N‐)glycosylated receptor, as would be the case for LRP‐1 and EGFR.

The NF‐κB pathway can be activated by various stimuli and its components can be involved in different signaling pathways depending on the stimulus, so it may even promote cell proliferation but not induce cell death as reported by other authors after stimulation with bLF, which could also be due to the deglycosylation of this glycoprotein. These results partially agree with previous data showing that bLF inhibited the phosphorylation of AKT^Ser473^ and NF‐κB p65 in stomach cancer cells (Xu et al., 2010). It has also been proposed that bLF positively interacts with the p53 (tumor suppressor protein) pathway that stabilizes the genome, regulates the cell cycle, and signals cell death apoptosis and ferroptosis (Kosim et al., 2022).

## 5 CONCLUSION

This work has shown that the glycans of bLF are directly related to its anticancer activity, and are responsible for cellular recognition, binding to the cell surface receptors. The stimulation of signaling pathways such as EGFR was different in HeLa than in Caco‐2 cells. In HeLa, phosphorylation of EGFR increased, possibly through stimulation via the LRP‐1 receptor, while bLF suppressed the ERK/Akt pathway in Caco2. Deglycosylation of bLF affected its binding and internalization and abolished the inhibitory activity on cancer cells, which was also seen in the signaling pathways. Future studies should address the full characterization of LF glycosylation sites with the stimulation/inhibition of signaling pathways leading to the inhibitory effect on cancer cells and its possible relevance in other beneficial roles attributed to bLF, such as antimicrobial, antiviral, or antifungal activity.

## AUTHOR CONTRIBUTIONS

**Diana A. Ramírez‐Sánchez:** Conceptualization (equal); formal analysis (lead); investigation (equal); methodology (equal); writing – original draft (equal). **Adrián Canizalez‐Román:** Conceptualization (equal); investigation (equal); supervision (equal). **Nidia León‐Sicairos:** Conceptualization (equal); investigation (equal); supervision (equal). **Gaspar Pérez Martínez:** Conceptualization (equal); formal analysis (equal); investigation (lead); methodology (equal); project administration (lead); resources (lead); supervision (lead); writing – original draft (lead); writing – review and editing (lead).

## Supporting information


**Figure S1.** Deglycosylation of cellular surfaces reduced binding of bLF to cells.

## Data Availability

Data are available on request from the authors. The data that support the findings of this study are available from the corresponding author upon reasonable request.
