# Supplementary material for: The anticancer activity of bovine lactoferrin is reduced by deglycosylation and it follows a different pathway in cervix and colon cancer cells
Source: Food Sci Nutr. 2024 Mar 7;12(5):3516–28. doi: 10.1002/fsn3.4020 (PMC11077203; doi:10.1002/fsn3.4020)
Supplement: Supplementary file 1 — Figure S1. Deglycosylation of cellular surfaces reduced binding of bLF to cells. [file FSN3-12-3516-s001.pptx]

## Slide 1
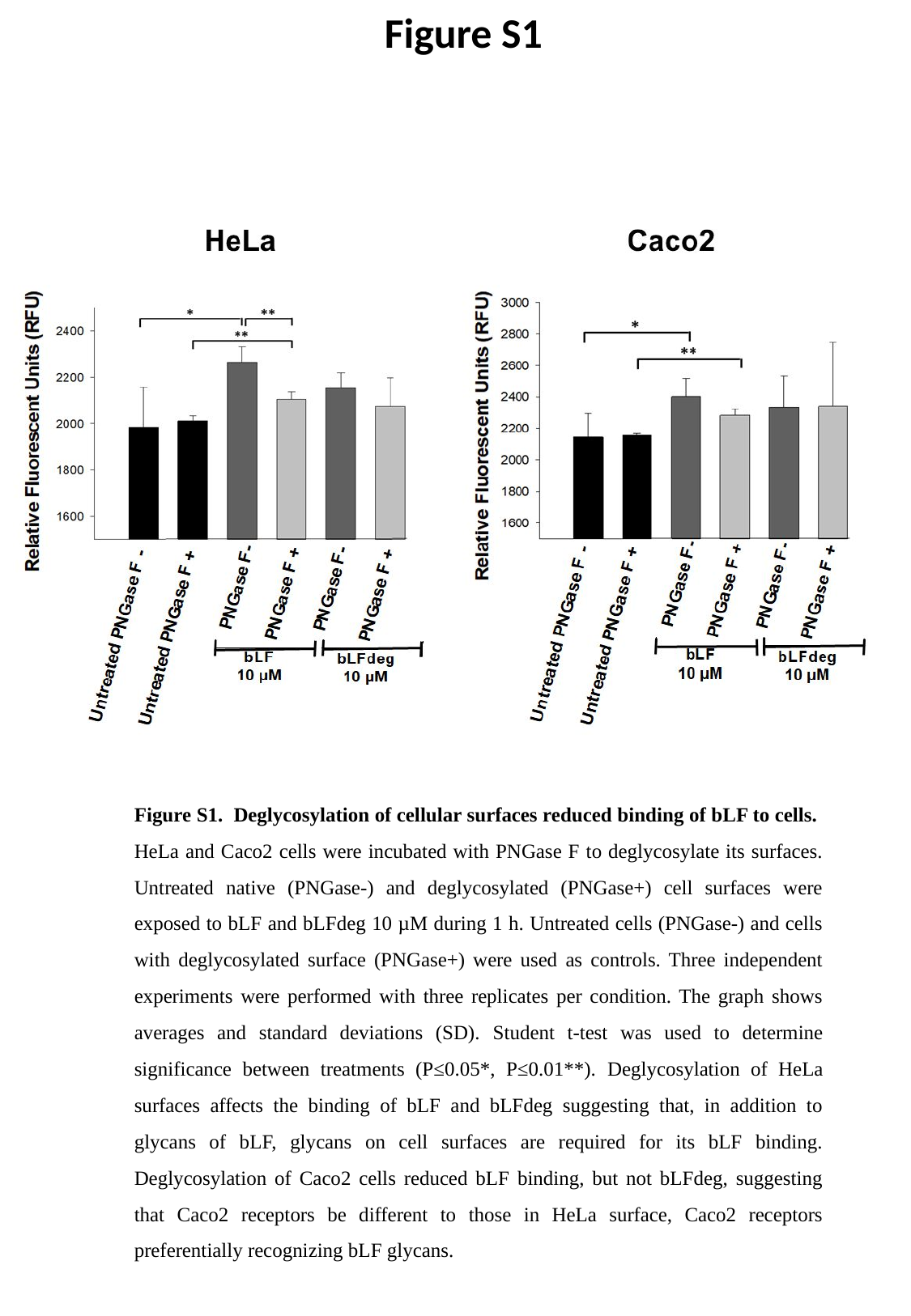

Figure S1
Figure S1. Deglycosylation of cellular surfaces reduced binding of bLF to cells. HeLa and Caco2 cells were incubated with PNGase F to deglycosylate its surfaces. Untreated native (PNGase-) and deglycosylated (PNGase+) cell surfaces were exposed to bLF and bLFdeg 10 µM during 1 h. Untreated cells (PNGase-) and cells with deglycosylated surface (PNGase+) were used as controls. Three independent experiments were performed with three replicates per condition. The graph shows averages and standard deviations (SD). Student t-test was used to determine significance between treatments (P≤0.05*, P≤0.01**). Deglycosylation of HeLa surfaces affects the binding of bLF and bLFdeg suggesting that, in addition to glycans of bLF, glycans on cell surfaces are required for its bLF binding. Deglycosylation of Caco2 cells reduced bLF binding, but not bLFdeg, suggesting that Caco2 receptors be different to those in HeLa surface, Caco2 receptors preferentially recognizing bLF glycans.
